# Perinatal death and exposure to dental amalgam fillings during pregnancy in the population-based MoBa cohort

**DOI:** 10.1371/journal.pone.0208803

**Published:** 2018-12-07

**Authors:** Lars Björkman, Gunvor B. Lygre, Kjell Haug, Rolv Skjærven

**Affiliations:** 1 Dental Biomaterials Adverse Reaction Unit, NORCE Norwegian Research Centre AS, Årstadveien, Bergen, Norway; 2 Department of Clinical Dentistry, University of Bergen, Bergen, Norway; 3 Department of Global Public Health and Primary Care, University of Bergen, Bergen, Norway; 4 Medical Birth Registry of Norway, Norwegian Institute of Public Health, Bergen, Norway; Medicina Fetal Mexico, MEXICO

## Abstract

**Objectives:**

The aim was to gain knowledge regarding the risk of perinatal death related to exposure to dental amalgam fillings in the mother.

**Design:**

Population-based observational cohort study.

**Setting:**

The Norwegian Mother and Child Cohort Study, a Norwegian birth cohort of children born in 1999–2008 conducted by the Norwegian Institute of Public Health.

**Participants:**

72,038 pregnant women with data on the number of teeth filled with dental amalgam.

**Main outcome measures:**

Data on perinatal death (stillbirth ≥ 22 weeks plus early neonatal death 0–7 days after birth) were obtained from the Medical Birth Registry of Norway.

**Results:**

The absolute risk of perinatal death ranged from 0.20% in women with no amalgam-filled teeth to 0.67% in women with 13 or more teeth filled with amalgam. Analyses including the number of teeth filled with amalgam as a continuous variable indicated an increased risk of perinatal death by increasing number of teeth filled with dental amalgam (crude OR 1.065, 95% CI 1.034 to 1.098, p<0.001). After adjustment for potential confounders (mothers' age, education, body mass index, parity, smoking during pregnancy, alcohol consumption during pregnancy) included as categorical variables, there was still an increased risk for perinatal death associated with increasing number of teeth filled with amalgam (OR_adj_ 1.041, 95% CI 1.008 to 1.076, p = 0.015). By an increased exposure from 0 to 16 teeth filled with amalgam, the model predicted an almost doubled odds ratio (OR_adj_ 1.915, 95% CI 1.12 to 3.28). In groups with 1 to 12 teeth filled with amalgam the adjusted odds ratios were slightly, but not significantly, increased. The group with the highest exposure (participants with 13 or more teeth filled with amalgam) had an adjusted OR of 2.34 (95% CI 1.27 to 4.32; p = 0.007).

**Conclusion:**

The current findings suggest that the risk of perinatal death could increase in a dose-dependent way based on the mother’s number of teeth filled with dental amalgam. However, we cannot exclude that the relatively modest odds ratios could be a result of residual confounding. Additional studies on the relationship between exposure to dental amalgam fillings during pregnancy and perinatal death are warranted.

## Introduction

Perinatal death of a child is associated with major emotional and social effects on the mother [1], and epidemiological information regarding maternal lifestyle exposure associated with stillbirth is a defined research priority. Maternal smoking, overweight and obesity are potentially modifiable established risk factors for stillbirth in high-income countries, but a large proportion of stillbirths is unexplained [2]. In high-income countries, a decrease in stillbirths has been observed over the past decades, but there is still considerable variation. Data indicate that further reductions in stillbirth are possible in high-income countries [2].

Dental amalgam has been used to treat caries lesions for more than 150 years [3, 4]. Amalgam fillings are durable and cost-effective compared with other types of restorations [5]. The main disadvantages of dental amalgam fillings are that they contain mercury, which is released as mercury vapour and inhaled [6–8], and that amalgam is not tooth coloured, and thus is less aesthetic. The use of amalgam has decreased in favour of tooth-coloured polymer-based composite materials [9]. There are also environmental concerns regarding the use of amalgam. In Norway the use of mercury for dental restoration was terminated in 2008, but in some other European countries the use continues at relatively high levels [10, 11]. Recently the European Parliament adopted new legislation aiming to phase out the use of mercury in dental amalgam by 2030 [12].

Amalgam fillings continuously release mercury as elemental vapour, which is absorbed into blood and distributed to body organs including the brain and the fetus [13–15], but there is no strong evidence that dental amalgam is a causal factor for disease [16, 17]. However, mercury has well-known detrimental effects on the fetus and exposure to methylmercury during pregnancy may cause severe effects on the fetus [17]. Currently there is only weak evidence that mercury vapour or inorganic mercury cause adverse effects on the fetus [18, 19]. There is, however, some evidence of increased risk of stillbirth or neonatal mortality in women with occupational exposure to amalgam and mercury. Naimi-Akbar et al reported increased risk of neonatal mortality for sons of dental nurses during the 1960s and a consistent decrease of the risk in the following decades when the exposure was lower [20–22]. An increased risk of miscarriage in dental personnel with moderate and high occupational exposure to amalgam was found in a Finnish study [23], and higher frequency than expected of spontaneous abortion and perinatal mortality was reported in a study of female dentists [24]. In a previous study of pregnancy outcomes related to dental amalgam exposure in the Norwegian Mother and Child Cohort Study (MoBa), the adjusted odds ratio (OR) for stillbirth was 1.38 (95%CI 0.80 to 2.39; p = 0.13) for mothers in the highest exposure group (9 or more teeth filled with dental amalgam) [25]. However, since the exposure variable was included as a categorical variable and not as a continuous variable, the power of the analysis was limited [26]. The aim of the present study was to gain knowledge about the associations between exposure to dental amalgam fillings in pregnant women and the risk of perinatal death (*i*.*e*. stillbirth plus early neonatal deaths 0–7 days post-partum).

## Materials and methods

### Data source and study cohort

MoBa is a prospective population-based pregnancy cohort study conducted by the Norwegian Institute of Public Health [27]. The aim is to gain knowledge on causes of diseases by estimating exposure before and after birth and the associations with outcomes among the children [28]. From 1999 to 2008 pregnant women from all over Norway were invited to participate in conjunction with the appointment for the routine ultrasound scan around pregnancy week 17. There were no exclusion criteria, but to be included the mothers had to be able to read Norwegian. The women consented to participation in 41% of the pregnancies. The cohort includes 114,500 children and 95,200 mothers, and each woman could contribute with more than one pregnancy. Follow-up is conducted by questionnaires at regular intervals and by linkage to national health registries [28]. The current study is based on version 8 of the quality-assured MoBa data files released for research on exposure to dental restorative materials during pregnancy and pregnancy outcomes.

The number of teeth present in the mouth and the number of teeth filled with dental amalgam were retrieved from questionnaire Q3, which was mailed to the participants during pregnancy week 30. The participants were asked to look in the mirror and count the number of teeth and the number of teeth filled with amalgam. Participants who had reported 32 or fewer teeth present in the mouth and an equal or lower number of teeth filled with dental amalgam were included in the analyses. Self-reporting of number of teeth and number of teeth filled with amalgam was previously validated and found to be reliable [29].

Information on current smoking habits, alcohol consumption and education was retrieved from questionnaire Q1, which was mailed to the participants in pregnancy week 17. Body mass index (BMI) was calculated from data at the start of the pregnancy obtained from Q1. Participants with height or weight coded with less probable values (height less than 140 cm or more than 200 cm, weight coded less than 40 kg or more than 200 kg) were given the code *missing* for the BMI variable. Level of education (fulfilled education of the mother at the start of pregnancy) was retrieved from questionnaire Q1.

Maternal age, birth weight, length of gestation at delivery, and parity were retrieved from the Medical Birth Registry of Norway (MBRN). Parity was coded “null” or “multi” and represented the number of previous births. The outcome variable “perinatal death” (stillbirth ≥ 22 weeks plus early neonatal death 0–7 days after birth) was retrieved from MBRN.

### Data analyses

Only singleton births were included in the analyses. Crude and adjusted ORs were calculated using logistic regression analysis (STATA, version 14.2) with adjustment of the standard errors for the clustering of mothers who participated with more than one pregnancy. Potential confounders were controlled for by inclusion in the models as categorical variables. Missing data within a variable were given the code *missing* and included in the analyses as a category within the actual variable. To explore changes over time in the exposure to dental amalgam in the cohort, the mean number of teeth filled with amalgam was calculated by year of inclusion in the cohort.

### Sensitivity analyses

To check the sensitivity of the model, restricted samples were analysed. Restrictions were made for the number of teeth filled with amalgam (exclusion of participants with more than 20 teeth filled with amalgam), very short pregnancies (model including only pregnancies longer than 30 weeks), children with malformations, children small for gestational age, and mothers with preeclampsia/eclampsia, chronic hypertension, and gestational diabetes.

### Patient involvement

The design of the MoBa study, the recruitment procedures, the aims of the study, and the research questions were discussed during the 1990's with the Norwegian Society for Gynecology and Obstetrics, international colleagues, and at national meetings with midwives. The study was also discussed in media and in the Norwegian Parliament before it became a national study. Potential participants or lay people were not involved in the design of the study, the development of the research questions or outcome measures, recruitment to or conduct of the study. The participants of the MoBa study are informed about the use of data, and the outcome of the research, through MoBa’s internet page (https://www.fhi.no/en/studies/moba/) and newsletters.

### Ethical approval

The establishment and data collection in MoBa has obtained a licence from the Norwegian Data Inspectorate and approval from The Regional Committee for Medical Research Ethics. Informed consent (https://www.fhi.no/globalassets/dokumenterfiler/studier/moba/dokumenter/samtykkeerklaring-mor.pdf) was obtained from each MoBa participant upon recruitment. The informed consent included consent to merge questionnaire data with data from the Medical Birth Registry of Norway (MBRN). The current study was approved by The Regional Committee for Medical Research Ethics in South-Eastern Norway (REC south-east D, number 2011/727). The data set was anonymized by the MoBa-study office before it was sent to us.

## Results

**Fig 1:**
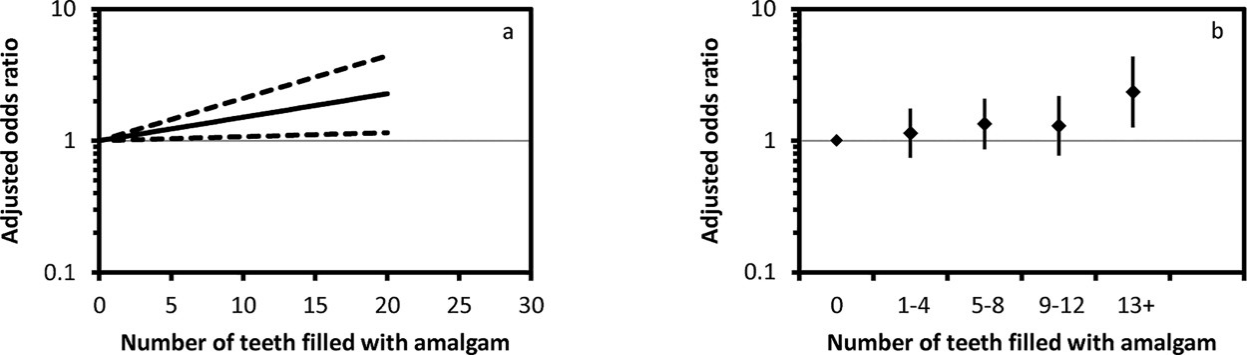
Adjusted odds ratio and 95% confidence interval by number of teeth filled with amalgam. Continuous exposure measure (a) and by exposure category (b).

The analyses included 72,038 singleton pregnancies (62,832 mothers participating with one to four pregnancies) with data on the number of teeth filled with amalgam. Descriptive data by amalgam group (0, 1–4, 5–8, 9–12, and 13+ teeth filled with amalgam) are given in Table 1. Of the included pregnancies, 208 (0.3%) ended with perinatal death (Table 2). The absolute risk for perinatal death ranged from 0.20% in participants with no amalgam-filled teeth to 0.67% in participants with 13 or more teeth filled with amalgam. Analyses including number of teeth filled with amalgam as a continuous variable indicated an increased risk of perinatal death for higher numbers of teeth filled with dental amalgam (crude OR 1.065, 95% CI 1.034 to 1.098, p<0.001, Table 3). After adjustment for potential confounders (mothers' age, education, body mass index, parity, smoking during pregnancy, alcohol consumption during pregnancy), the adjusted OR was 1.041 (95% CI 1.008 to 1.076, p = 0.015, Table 3), indicating an increased risk for perinatal death associated with higher numbers of teeth filled with amalgam. The OR was almost doubled (OR 1.915, 95% CI 1.12 to 3.28) for participants with 16 teeth filled with amalgam compared with participants without amalgam fillings (Fig 1). There was no major effect from clustering of pregnancies within mothers.

**Table 1:** Descriptive data of the study cohort by amalgam group.

		Amalgam group											
		0		1–4		5–8		9–12		13+		Total	
		n	%	n	%	n	%	n	%	n	%	n	%
Number of pregnancies		15699	21.8%^a^	26031	36.1%^a^	19072	26.5%^a^	8685	12.1%^a^	2551	3.5%^a^	72038	100.0%^a^
Mother’s age (years)	-19	288	1.8%	185	0.7%	50	0.3%	6	0.1%	1	0.04%	530	0.7%
	20–24	2585	16.5%	2451	9.4%	1023	5.4%	255	2.9%	63	2.5%	6377	8.9%
	25–29	6574	41.9%	9388	36.1%	5392	28.3%	1995	23.0%	490	19.2%	23839	33.1%
	30–34	5061	32.2%	10514	40.4%	8238	43.2%	3710	42.7%	1000	39.2%	28523	39.6%
	35–39	1103	7.0%	3213	12.3%	3872	20.3%	2334	26.9%	773	30.3%	11295	15.7%
	40+	88	0.6%	280	1.1%	497	2.6%	385	4.4%	224	8.8%	1474	2.0%
Parity	Null	8963	57.1%	12327	47.4%	7488	39.3%	2864	33.0%	776	30.4%	32418	45.0%
	One or more	6736	42.9%	13704	52.6%	11584	60.7%	5821	67.0%	1775	69.6%	39620	55.0%
Body mass index (kg/m^2^)	<18.5	537	3.4%	817	3.1%	456	2.4%	156	1.8%	44	1.7%	2010	2.8%
	18.5–25	10679	68.0%	17285	66.4%	11684	61.3%	4946	56.9%	1334	52.3%	45928	63.8%
	25–30	2959	18.8%	5102	19.6%	4331	22.7%	2216	25.5%	695	27.2%	15303	21.2%
	30–35	822	5.2%	1512	5.8%	1432	7.5%	732	8.4%	261	10.2%	4759	6.6%
	35–40	207	1.3%	422	1.6%	353	1.9%	251	2.9%	78	3.1%	1311	1.8%
	>40	64	0.4%	122	0.5%	126	0.7%	81	0.9%	35	1.4%	428	0.6%
	Missing	431	2.7%	771	3.0%	690	3.6%	303	3.5%	104	4.1%	2299	3.2%
Education (years)	-12	4493	28.6%	7501	28.8%	6240	32.7%	3283	37.8%	1043	40.9%	22560	31.3%
	13–16	6210	39.6%	10625	40.8%	7663	40.2%	3434	39.5%	946	37.1%	28878	40.1%
	17+	3932	25.0%	6405	24.6%	4108	21.5%	1534	17.7%	427	16.7%	16406	22.8%
	Missing	1064	6.8%	1500	5.8%	1061	5.6%	434	5.0%	135	5.3%	4194	5.8%
Current smoking	Non smoker	13395	85.3%	21331	81.9%	14679	77.0%	6327	72.8%	1721	67.5%	57453	79.8%
	Occasionally	338	2.2%	609	2.3%	506	2.7%	283	3.3%	80	3.1%	1816	2.5%
	Daily smoking	474	3.0%	1047	4.0%	1103	5.8%	614	7.1%	231	9.1%	3469	4.8%
	Missing	1492	9.5%	3044	11.7%	2784	14.6%	1461	16.8%	519	20.3%	9300	12.9%
Use of alcohol	Never	12238	78.0%	19488	74.9%	13834	72.5%	6047	69.6%	1688	66.2%	53295	74.0%
	1–3 times per month or less	1290	8.2%	2618	10.1%	2289	12.0%	1171	13.5%	384	15.1%	7752	10.8%
	1 time per week or more	51	0.3%	113	0.4%	113	0.6%	65	0.7%	18	0.7%	360	0.5%
	Missing	2120	13.5%	3812	14.6%	2836	14.9%	1402	16.1%	461	18.1%	10631	14.8%

^a^ Row%

**Table 2:** Number of cases of perinatal death related to the mother’s number of teeth filled with amalgam.

			Amalgam group					Total
	Perinatal death		0	1–4	5–8	9–12	13+	
Entire study cohort	Yes	n (%)	32 (0.20)	66 (0.25)	63 (0.33)	30 (0.35)	17 (0.67)	208 (0.29)
	No	n	15,667	25,965	19,009	8,655	2,534	71,830
First cohort (1999–2003)^a^	Yes	n (%)	5 (0.17)	20 (0.29)	20 (0.28)	13 (0.33)	11 (0.80)	69 (0.31)
	No	n	2,960	6,955	7,088	3,960	1,368	22,331
Second cohort (2004–2008)^a^	Yes	n (%)	27 (0.22)	42 (0.23)	42 (0.37)	17 (0.38)	6 (0.55)	134 (0.28)
	No	n	12,332	18,292	11,374	4,452	1,085	47,535

Data given for the entire study cohort, the first cohort (included between 1999 and 2003), and the second cohort (included between 2004 and 2008).

^a^ Data regarding cohort missing for 1,969 observations.

**Table 3:** Results from analyses of all pregnancies (n = 72,038) compared with restricted samples.

Model	Total (n)	Cases (n)	OR _crude_ (95% CI)	p-value	OR _adj_ (95% CI) ^a^	p-value
No restrictions (all pregnancies)	72,038	208	1.065 (1.034 to 1.098)	<0.001	1.041 (1.008 to 1.076)	0.015
Mothers with maximum 20 teeth filled with amalgam	71,966	208	1.069 (1.036 to 1.103)	<0.001	1.044 (1.009 to 1.081)	0.013
Pregnancies longer than 30 weeks	71,593	197	1.064 (1.031 to 1.098)	<0.001	1.039 (1.004 to 1.075)	0.030
Children with malformations excluded	68,599	172	1.056 (1.020 to 1.093)	0.002	1.035 (0.996 to 1.075)	0.075
Children small for gestational age excluded	67,055	131	1.086 (1.047 to 1.128)	<0.001	1.063 (1.019 to 1.107) ^b^	0.004
Mothers with preeclampsia / eclampsia excluded	69,488	198	1.070 (1.038 to 1.103)	<0.001	1.047 (1.012 to 1.082)	0.008
Mothers with chronic hypertension excluded	71,668	205	1.065 (1.033 to 1.098)	<0.001	1.042 (1.008 to 1.077)	0.016
Mothers with gestational diabetes excluded	71,458	208	1.066 (1.034 to 1.098)	<0.001	1.041 (1.008 to 1.076)	0.016

Crude and adjusted odds ratios (and 95% confidence intervals) are given for associations between number of teeth filled with amalgam (as a continuous variable) and perinatal death.

^a^ Adjusted for mother’s age, education, BMI, parity, smoking, alcohol intake

^b^ n = 66,718; 337 observations dropped in the analysis because of missing cell-information

Results from analysis by exposure group (0, 1–4, 5–8, 9–12, and 13+ teeth filled with amalgam) are given in Table 4. For the highest exposure group (participants with 13 or more teeth filled with amalgam) the adjusted OR was 2.341 (95% CI 1.267 to 4.324; p = 0.007, Fig 1).

**Table 4:** Crude and adjusted odds ratios (and 95% confidence intervals; CI).

		Amalgam group																																			
Sample	Model	0	1–4							5–8							9–12							13+							Continuous variable						
		OR	OR		(95%CI)				p-value	OR		(95%CI)				p-value	OR		(95%CI)				p-value	OR		(95%CI)				p-value	OR		(95%CI)				p-value
All pregnancies	Crude (n = 72,038)	1 (reference)	1.24	(	0.82	to	1.90	)	0.310	1.62	(	1.06	to	2.48	)	0.026	1.70	(	1.03	to	2.79	)	0.038	3.28	(	1.82	to	5.92	)	<0.001	1.065	(	1.034	to	1.098	)	<0.001
	Adjusted^a^ (n = 72,038)	1 (reference)	1.14	(	0.74	to	1.75	)	0.562	1.34	(	0.87	to	2.06	)	0.188	1.29	(	0.76	to	2.20	)	0.342	2.34	(	1.27	to	4.32	)	0.007	1.041	(	1.008	to	1.076	)	0.015
Non-smokers	Crude (n = 57,453)	1 (reference)	1.21	(	0.74	to	1.96	)	0.448	1.64	(	1.01	to	2.68	)	0.046	2.29	(	1.33	to	3.95	)	0.003	2.50	(	1.13	to	5.54	)	0.024	1.072	(	1.036	to	1.109	)	<0.001
	Adjusted^b^ (n = 57,453)	1 (reference)	1.13	(	0.69	to	1.85	)	0.622	1.46	(	0.89	to	2.38	)	0.135	1.95	(	1.10	to	3.44	)	0.022	2.07	(	0.91	to	4.73	)	0.083	1.059	(	1.021	to	1.099	)	0.002
Smokers	Crude (n = 5,285)	1 (reference)	0.98	(	0.18	to	5.37	)	0.982	3.30	(	0.74	to	14.66	)	0.117	0.45	(	0.04	to	5.00	)	0.517	3.94	(	0.66	to	23.73	)	0.134	1.066	(	0.990	to	1.148	)	0.091
	Adjusted^b^ (n = 4,252*)	1 (reference)	0.78	(	0.15	to	4.03	)	0.771	2.06	(	0.49	to	8.72	)	0.325	0.23	(	0.02	to	2.41	)	0.222	1.76	(	0.29	to	10.81	)	0.539	1.013	(	0.934	to	1.098	)	0.758
Normal body weight index (BMI 18.5–24.9 kg/m^2^)	Crude (n = 45,928)	1 (reference)	0.93	(	0.55	to	1.55	)	0.772	1.10	(	0.64	to	1.90	)	0.244	0.99	(	0.48	to	2.02	)	0.977	2.68	(	1.20	to	5.97	)	0.016	1.036	(	0.987	to	1.087	)	0.156
	Adjusted^c^ (n = 45,928)	1 (reference)	0.88	(	0.52	to	1.51	)	0.648	1.01	(	0.57	to	1.80	)	0.965	0.88	(	0.41	to	1.88	)	0.737	2.31	(	1.00	to	5.30	)	0.049	1.025	(	0.974	to	1.080	)	0.346
Overweight/Obese (BMI over 25 kg/m^2^)	Crude (n = 21,801)	1 (reference)	1.70	(	0.76	to	3.79	)	0.194	2.60	(	1.20	to	5.66	)	0.016	2.95	(	1.29	to	6.74	)	0.011	2.85	(	0.99	to	8.23	)	0.052	1.077	(	1.036	to	1.120	)	<0.001
	Adjusted^c^ (n = 21,618*)	1 (reference)	1.52	(	0.68	to	3.39	)	0.312	2.16	(	1.01	to	4.61	)	0.048	2.30	(	0.97	to	5.42	)	0.058	2.18	(	0.76	to	6.28	)	0.147	1.060	(	1.016	to	1.105	)	0.007
Low education (12 years or less)	Crude (n = 22,560)	1 (reference)	2.40	(	0.80	to	7.18	)	0.118	4.33	(	1.50	to	12.50	)	0.007	2.74	(	0.82	to	9.11	)	0.100	7.58	(	2.22	to	25.94	)	0.001	1.094	(	1.046	to	1.144	)	<0.001
	Adjusted^d^ (n = 22,482*)	1 (reference)	1.97	(	0.64	to	6.08	)	0.239	2.92	(	0.98	to	8.70	)	0.055	1.65	(	0.46	to	6.00	)	0.444	4.28	(	1.15	to	15.89	)	0.030	1.056	(	0.999	to	1.115	)	0.054
High education (more than 12 years)	Crude (n = 45,284)	1 (reference)	1.01	(	0.63	to	1.63	)	0.952	1.12	(	0.68	to	1.85	)	0.665	1.44	(	0.80	to	2.59	)	0.226	2.75	(	1.33	to	5.68	)	0.006	1.051	(	1.007	to	1.096	)	0.022
	Adjusted^d^ (n = 45,112*)	1 (reference)	0.97	(	0.60	to	1.57	)	0.905	0.99	(	0.59	to	1.65	)	0.968	1.18	(	0.64	to	2.16	)	0.595	2.08	(	0.99	to	4.38	)	0.053	1.031	(	0.987	to	1.077	)	0.172
Boys	Crude (n = 36,826)	1 (reference)	0.88	(	0.49	to	1.58	)	0.680	1.44	(	0.82	to	2.54	)	0.203	1.34	(	0.67	to	2.68	)	0.405	2.63	(	1.15	to	6.02	)	0.022	1.061	(	1.015	to	1.109	)	0.009
	Adjusted^a^ (n = 36,537*)	1 (reference)	0.79	(	0.44	to	1.42	)	0.428	1.13	(	0.64	to	1.98	)	0.678	0.97	(	0.46	to	2.01	)	0.925	1.74	(	0.74	to	4.08	)	0.204	1.032	(	0.983	to	1.083	)	0.202
Girls	Crude (n = 35,207)	1 (reference)	1.73	(	0.92	to	3.25	)	0.090	1.89	(	0.98	to	3.62	)	0.056	2.22	(	1.06	to	4.61	)	0.033	4.23	(	1.81	to	9.91	)	0.001	1.071	(	1.028	to	1.116	)	0.001
	Adjusted^a^ (n = 35,207)	1 (reference)	1.62	(	0.85	to	3.11	)	0.146	1.65	(	0.84	to	3.21	)	0.144	1.79	(	0.82	to	3.90	)	0.143	3.25	(	1.33	to	7.96	)	0.010	1.053	(	1.007	to	1.101	)	0.024

Data given for analyses of associations between number of teeth filled with dental amalgam (included as categories or as a continuous variable) and perinatal death in the total sample and in subgroups.

^a^ Adjusted for parity, BMI, mother’s age, education, smoking, alcohol intake

^b^ Adjusted for parity, BMI, mother’s age, education, alcohol intake

^c^ Adjusted for parity, mother’s age, education, smoking, alcohol intake

^d^ Adjusted for parity, BMI, mother’s age, smoking, alcohol intake

* Cases dropped in the analysis because of collinearity or missing cell-information

### Sensitivity analyses

In a restricted sample excluding mothers with more than 20 teeth filled with amalgam, the adjusted OR was 1.044 (95% CI 1.009 to 1.081, p = 0.013; Table 3). Results from analyses of samples excluding pregnancies 30 weeks or shorter, children with malformations, and mothers with preeclampsia/eclampsia, chronic hypertension, and gestational diabetes, showed generally similar, and consistent, results (Table 3). When children small for gestational age were excluded, the adjusted OR was 1.063 (95% CI 1.019 to 1.107, p = 0.004; Table 3) for the continuous measure. For participants with 13 or more teeth filled with amalgam, the adjusted OR was 2.847 (95% CI 1.356 to 5.976; p = 0.006).

### Analyses of subgroups

Non-smokers who had 13 or more teeth filled with amalgam had an adjusted OR of 2.074 (95% CI from 0.910 to 4.728, p = 0.083, Table 4). The adjusted OR using the continuous exposure measure was higher for non-smokers analysed separately than for the entire cohort (OR_adj_ 1.059, 95% CI from 1.021 to 1.099, p = 0.002). For smoking mothers (n = 5,285) there was less evidence of an association. The adjusted OR using the continuous exposure measure was 1.013 (95% CI from 0.934 to 1.098, p = 0.758; Table 4).

When only participants with normal body mass index (BMI from 18.5 to 24.9 kg/m^2^) were included in the analysis, the adjusted OR for the continuous measure was 1.025 (95% CI 0.974 to 1.080, p = 0.346). For participants with BMI over 25 kg/m^2^ (overweight and obese), the adjusted OR was 1.060 (95% CI 1.016 to 1.105, p = 0.007).

In participants with low education (12 years or less) the adjusted OR was significantly elevated for those in the highest exposure group (OR_adj_ 4.28, 95% CI 1.15 to 15.89; p = 0.030). For participants with high education (more than 12 years) the adjusted OR for the highest exposure group was 2.08 (95% CI 0.99 to 4.38, p = 0.053). The association between the risk of perinatal death of the child and the mother’s exposure to dental amalgam was not statistically significant for participants with high education when the continuous exposure measure was used and adjusted for covariates (Table 4).

When girls and boys were analysed separately, there was evidence for an increased risk for girls, but not for boys. The adjusted OR for girls was 1.053 (95% CI 1.007 to 1.101, p = 0.024, Table 4). For boys the adjusted OR was 1.032 (95% CI 0.983 to 1.083, p = 0.203). For five pregnancies the sex of the child was unknown.

**Fig 2:**
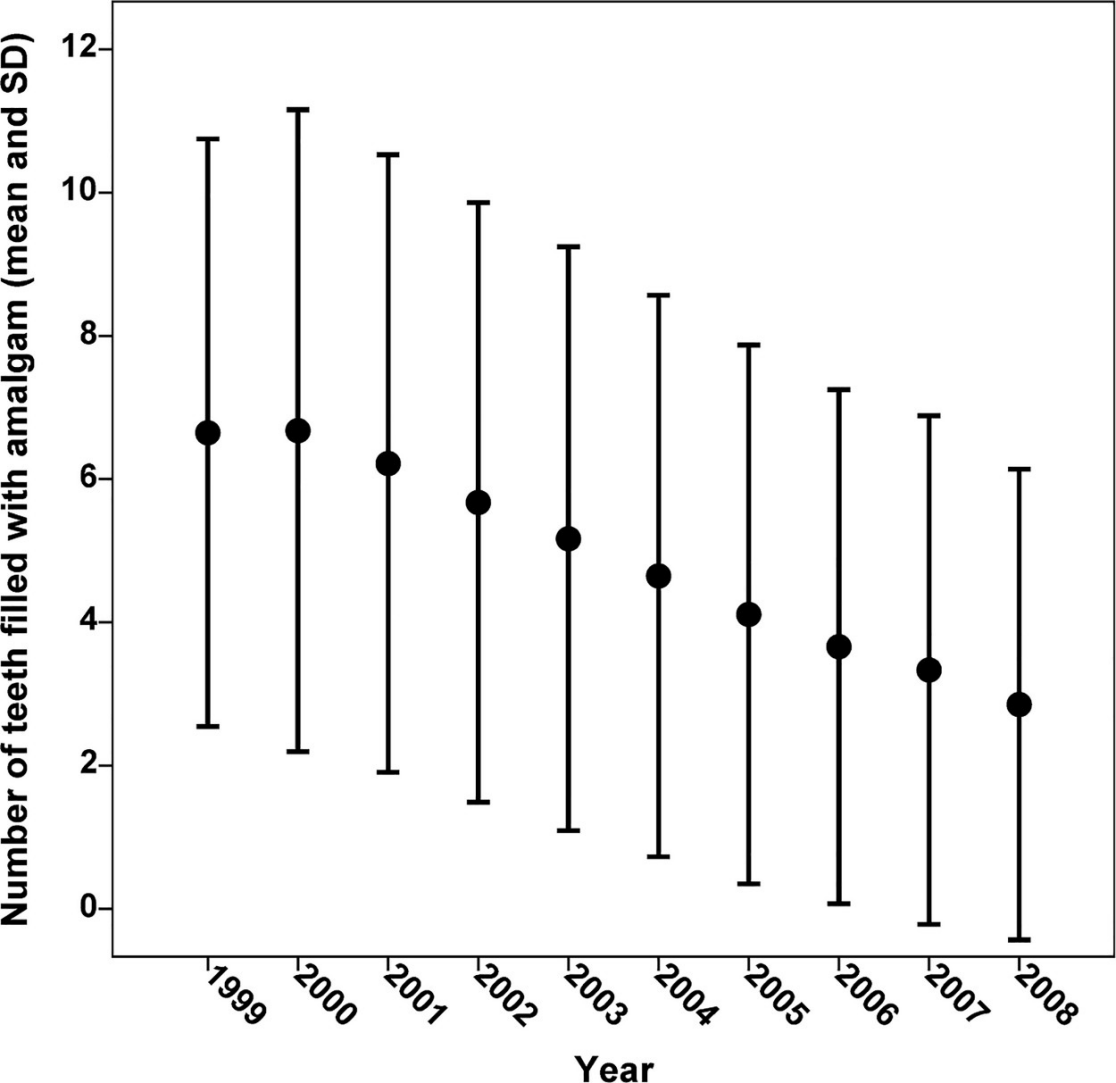
Number of teeth filled with amalgam (mean and standard deviation) in the study cohort by year (n = 70,069).

In the MoBa cohort the number of teeth filled with dental amalgam decreased considerably from the start of the inclusion period in 1999 to the end in 2008. The mean number of teeth filled with amalgam was 6.6 in 1999, while it was 2.9 in 2008 (Fig 2). Likewise, the proportion of participants with 13 or more teeth filled with amalgam decreased from 8.5% (25 of 295) in 1999 to 1.3% (85 of 6,321) in 2008.

The incidence of perinatal mortality decreased from 0.31% the first five years (1999 to 2003) to 0.28% the last five years (2004 to 2008, Table 2). The decrease of the absolute risk 0.03% (*i*.*e*. the absolute risk reduction) was, however, not statistically significant (p = 0.549; Fisher’s exact test).

## Discussion

### Key results

The main finding was the statistically significant association between the number of teeth filled with dental amalgam and the risk of perinatal death. Adjustment for potential confounders changed the risk estimate slightly, but it was still statistically significant after adjustment. The results are consistent with those from analyses of the same cohort regarding stillbirth [25]. In the present study both stillbirths and deaths in the first week after birth were included. Thus, more cases were available for the analyses. In addition, the use of the number of teeth filled with amalgam as a continuous variable and considering clustering by participants who were included more than once in the data set increased the power of the analyses.

### Comparison with other studies

**Fig 3:**
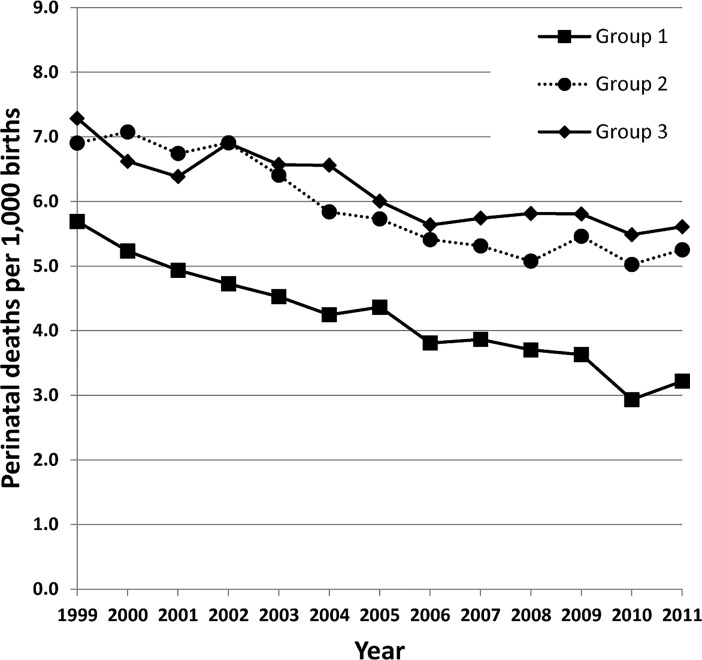
Mean perinatal mortality (deaths per 1,000 births) by year for group 1, 2 and 3 countries (see text and S1 Table). Data from Bio Intelligence Service and World Health Organization Regional Office for Europe.

The result is supported by results from studies of individuals with occupational exposure to dental amalgam related to neonatal death or miscarriage [20, 23, 24]. Other studies on occupational exposure to mercury vapour and risk of neonatal death or miscarriage have been inconclusive [30–32]. Dental personnel who work with amalgam have higher levels of mercury in the body [33–36], and individuals with amalgam fillings are continuously exposed to mercury as elemental mercury vapour and in corrosion products [8, 37–39]. Mercury released from amalgam fillings is absorbed into the blood and distributed to the body [13, 14], and inorganic mercury absorbed by pregnant women may pass to the fetus [40]. It has been shown that mercury concentrations in fetal and infant tissues correlate significantly with the number of dental amalgam fillings of the mother [15]. Since any protein may be a target for mercury [41], there is a potential for adverse effects on the fetus from exposure to mercury released from the mother's amalgam fillings. Consistent with the considerable decrease over time of the number of teeth filled with amalgam among participants in the MoBa cohort (Fig 2), the use of amalgam has decreased in most countries in the last decades in favour of other restorative materials. In parallel, perinatal death rates have decreased considerably in Norway [42]. In the period 1996–2000 the national perinatal mortality rate (stillbirths and deaths under one week per 1,000 births) was 6.3, while it was 5.5 in the period 2001–2005, and 5.0 in 2008 [43]. In the European Union there is considerable variation in the use of dental amalgam. In a report to the European Commission EU countries were divided into groups depending on the estimated proportion of amalgam fillings placed in 2010 (0–5%; Group 1, 6–35%; Group 2, and >35%; Group 3; S1 Table) [11]. The mean rate of perinatal deaths per 1,000 births (using data for 2011 from the European Health For All database; http://data.euro.who.int/hfadb/ [44]) in Group 1 countries was 3.2. In countries with a more frequent use of amalgam, the rates were higher (5.3 and 5.6, respectively). A gradual decrease in perinatal mortality was observed in all three groups from 1999 to 2011 (Fig 3). Even though these data may support the hypothesis of an association between the use of amalgam and the rate of perinatal death, there are considerable differences between the countries with respect to economy and health services, which may be significant.

### Subgroup analyses

The subgroup analyses showed consistent results with the main analysis. All confidence intervals of the subgroups included the OR for the entire cohort. For smokers analysed separately there was less evidence of an association between the number of filled teeth with amalgam and the risk of perinatal death, but the confidence interval was wide and included the OR for the entire cohort. The sensitivity analysis (Table 3) showed that when mothers who gave birth to SGA children were excluded, the adjusted OR was higher compared with the adjusted OR for the entire cohort, both for the continuous exposure measure and for participants with 13 or more teeth filled with amalgam. In our previous study [25], we found no evidence of an association between amalgam exposure and risk for giving birth to SGA children. Thus, the association between amalgam exposure and risk for perinatal death of the child is probably not mediated by growth restriction of the fetus.

### Strengths and limitations

The MoBa cohort is very large and the prospective design with data collection early in the pregnancy reduces the risk for recall bias. In addition, the exposure measure was validated in an earlier study and found to be reliable [29]. Since the results were similar if cases of perinatal death occurring in pregnancy week 30 or earlier were excluded, the risk of bias due to misclassification among women with very short pregnancies is assessed to be low. One of the main limitations of the study is that the incidence of perinatal death was low in the cohort (3 per 1,000 pregnancies; 0.3%), and thus there are few cases. Another limitation is the lack of a biological exposure indicator of inorganic mercury. Measurement of mercury concentration in urine could give additional useful information regarding the possible association between exposure to inorganic mercury and outcome. Dental amalgam is the dominating source of inorganic mercury in the population [45]. Even though there is a significant correlation between the number of tooth surfaces filled with amalgam and mercury concentration in urine [46, 47], there is considerable variation in exposure to mercury due to bruxism, chewing habits and diet [8, 37, 38]. Consequently, if exposure to inorganic mercury is a causal factor for perinatal deaths among women with dental amalgam fillings, the number of teeth filled with dental amalgam would be a proxy for exposure to inorganic mercury, and the risk associated with the actual exposure to mercury could be underestimated in the present analyses. Even though the exposure estimate was validated and was found to be reliable [29], there is some imprecision in the exposure estimate. The participants were instructed to look in the mirror and to count the number of teeth with amalgam fillings. Data from the validation study showed that agreement within a variation of ±1 tooth was found in 88% of data pairs [29]. Likewise, 87% were placed in the correct amalgam exposure group. We have no indications of a differential misclassification, but the imprecision may result in underestimation of the OR.

### Generalisability

Because of self-selection bias in the MoBa cohort, the cohort is not representative of the Norwegian population [48]. For instance, the frequencies of stillbirth and neonatal death were lower in the cohort compared to the general Norwegian population (Table 2) [48]. However, estimates of exposure-outcome associations found in the MoBa cohort may have acceptable external validity and be useful for generalisation [48]. Exposure to amalgam among pregnant women in Norway could be different between urban and rural regions because of differences in access to dental care. In addition, access to advanced neonatal hospital care could also be different between the regions, and could be a potential confounder in the analyses. Large hospitals in urban regions may have more resources for providing neonatal care, but they may also have the most complicated cases referred for specialty care. To address this issue, adjustment for hospital size (number of births per year) was made, but the results were similar.

### Interpretation and clinical implications

The results from this study support the hypothesis of increased risk of perinatal mortality of children born by women with many amalgam fillings. Mothers with more than 12 teeth filled with amalgam had an adjusted OR of 2.34. Using the advantage of the prospective cohort design and the OR as an estimate of the relative risk, we used a standard epidemiologic formula to calculate the attributable fraction [49]. We estimated that among mothers with more than 12 teeth filled with amalgam, 57% of the cases were attributable to amalgam. This estimate is based on the assumptions that there is a causal relationship and that the estimated adjusted OR is unbiased [49]. Since the OR was relatively modest, it cannot be ruled out that residual or unknown confounding could change the estimate. However, the association between perinatal mortality and exposure to amalgam is supported by studies on occupational exposed dental personnel. In addition, the gradual decrease over time in several countries of both the use of amalgam as restorative material and the incidence rates for perinatal mortality provides further support to the hypothesis of an association.

A global phase-down of the use of dental amalgam was decided at the UNEP conference in Minamata [50]. Thus far, more than 120 countries have signed the treaty (http://www.mercuryconvention.org/Countries). The recently adopted legislation by the European Parliament aiming at phasing out the use of mercury in dental amalgam by 2030 [12] will reduce the gap between the EU legislation and the United Nations Minamata Convention against mercury pollution [50]. As a preventive measure, exposure to all forms of mercury should be as low as possible [19, 45], and additional studies on the relationship between exposure to dental amalgam fillings during pregnancy and perinatal death are warranted.

## Supporting information

S1 File Data used for Fig 3. (PDF)

S2 File References to Questionnaire 1 and Questionnaire 3. (PDF)

S1 Table EU countries divided into groups of estimated proportion of amalgam fillings placed in 2010. (PDF)
