# Supplementary material for: Perinatal death and exposure to dental amalgam fillings during pregnancy in the population-based MoBa cohort
Source: PLoS One. 2018 Dec 7;13(12):e0208803. doi: 10.1371/journal.pone.0208803 (PMC6286137; doi:10.1371/journal.pone.0208803)
Supplement: S1 File — (PDF) [file pone.0208803.s001.pdf]

S1 File. Data used for Fig 3.

http://data.euro.who.int/hfadb/

Perinatal deaths per 1000 births

group	Years	group	1999	2000	2001	2002	2003	2004	2005	2006	2007	2008	2009	2010	2011
1	Denmark	1	6.22	5.8	5.48	4.84	4.85	4.7	4.82	3.77	4.12	3.47	2.83	3.03	2.88
1	Estonia	1	7.83	6.37	5.79	6	5.84	5.16	6.07	4.17	4.13	4.26	4.51	3.17	3.14
1	Finland	1	3.16	3.67	3.29	3.37	3.4	2.91	3.02	3.06	3.33	2.91	2.84	2.52	2.52
1	Italy	1	6.66	5.63	5.67	5.44	4.66	4.55	4.56	4.45	4.28	4.45	4.61	...	4.35
1	Sweden	1	4.57	4.68	4.44	3.96	3.87	3.9	3.34	3.59	3.46	3.42	3.35	3.01	3.2
Mean group 1			**5.688**	**5.230**	**4.934**	**4.722**	**4.524**	**4.244**	**4.362**	**3.808**	**3.864**	**3.702**	**3.628**	**2.933**	**3.218**
2	Belgium	2	4.87	5.01	4.55	4.57	4.61	4.42	4.12	4.18	4.21	4.01	3.96	4.06	4.04
2	Bulgaria	2	12.95	12.16	12.32	12.57	12.1	12.21	12.02	10.69	10.97	10.54	11.3	11.38	11.69
2	Cyprus (excluded)	2	...	...	...	...	...	...	...	...	x 4.19	...	...	...	x 4.87
2	Germany	2	3.91	6.07	5.94	5.83	5.91	5.89	5.55	5.55	5.52	5.3	5.33	5.35	5.47
2	Hungary	2	5.39	5.74	5.7	5.38	5.34	4.8	5.01	4.86	4.92	4.32	4.68	4.12	4.16
2	Ireland	2	5.95	6.29	6.06	6.29	6.31	5.54	5.25	5.05	5.19	4.8	5.11	4.79	4.21
2	Latvia	2	9.97	9.06	8.82	10.65	7.75	7.8	8.11	7.39	6.53	6.29	7.04	5.74	7.09
2	Luxembourg	2	5.36	6.78	4.49	4.8	4.5	4.16	3.46	2.98	3.21	3.65	4.54	3.38	3.97
2	Netherlands	2	9.46	9.05	9.19	8.83	8.34	5.04	5.21	4.62	4.3	4.19	4.27	4.07	4.13
2	Portugal	2	5.46	5.07	4.72	4.9	4.25	3.63	3.64	3.98	3.79	3.27	3.82	2.93	3.16
2	Spain	2	5.68	5.51	5.6	5.24	4.93	4.87	4.91	4.78	4.47	4.37	4.53	4.42	4.59
Mean group 2			**6.900**	**7.074**	**6.739**	**6.906**	**6.404**	**5.836**	**5.728**	**5.408**	**5.311**	**5.074**	**5.458**	**5.024**	**5.251**
3	Austria	3	3.79	3.87	3.55	3.43	3.61	3.2	3.38	3.2	3.1	2.96	3.02	3.01	3.09
3	Czech Republic	3	4.72	4.01	3.78	3.97	3.65	3.58	3.49	3.63	3.58	2.93	3.24	3.08	3.63
3	France	3	6.55	6.58	6.91	9.99	10.77	10.87	10.47	10.92	10.75	12.05	13.35	11.73	11.04
3	Greece	3	8.77	7.88	8	7.22	6.58	6.28	5.66	4.94	5.32	4.4	5.5	5.86	5.37
3	Lithuania	3	6.69	8.27	5.72	7.29	6.01	6.27	5.42	5.74	5.58	5.3	4.96	4.41	4.74
3	Malta	3	9.92	4.6	5.09	6.65	4.72	5.91	3.12	1.8	4.12	6.91	6.02	6.22	7.69
3	Poland	3	7.55	6.68	6.34	5.75	5.6	5.63	5.27	4.96	4.89	4.72	4.02	4.24	4.15
3	Romania	3	11.83	12.1	11.77	11.76	11.79	12.24	10.57	9.83	8.84	7.96	7.33	7.04	6.88
3	Slovakia	3	7.17	6.57	6.48	6.39	6.72	5.82	5.47	5.58	5.33	5.35	5.11	4.62	4.36
3	Slovenia	3	4.89	4.09	4.54	5.15	4.34	4.16	5.2	3.49	3.89	3.81	3.7	2.71	3.23
3	United Kingdom	3	8.23	8.15	8.02	8.3	8.46	8.16	7.95	7.89	7.72	7.54	7.6	7.39	7.48
Mean group 3			**7.283**	**6.618**	**6.382**	**6.900**	**6.568**	**6.556**	**6.000**	**5.635**	**5.738**	**5.812**	**5.805**	**5.483**	**5.605**

**Year**	**1999**	**2000**	**2001**	**2002**	**2003**	**2004**	**2005**	**2006**	**2007**	**2008**	**2009**	**2010**	**2011**
**Group 1**	5.688	5.230	4.934	4.722	4.524	4.244	4.362	3.808	3.864	3.702	3.628	2.933	3.218
**Group 2**	6.900	7.074	6.739	6.906	6.404	5.836	5.728	5.408	5.311	5.074	5.458	5.024	5.251
**Group 3**	7.283	6.618	6.382	6.900	6.568	6.556	6.000	5.635	5.738	5.812	5.805	5.483	5.605

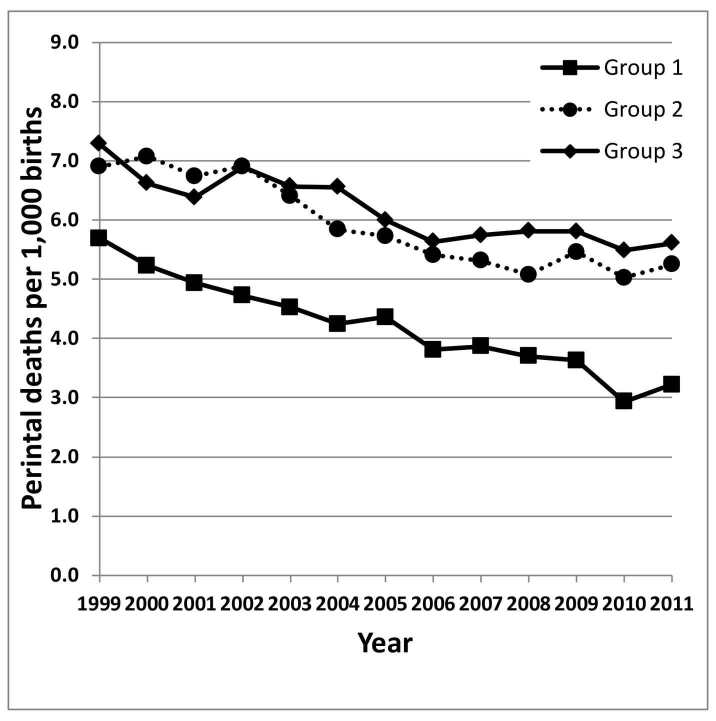

DATA FROM: Bio Intelligence Service. Study on the potential for reducing mercury pollution from dental amalgam and batteries, Final report prepared for the European Commission – DG ENV. Paris: Bio Intelligence Service, 2012.

Table 4-k Projected demand for mercury in dental amalgam
http://eur-lex.europa.eu/legal-content/EN/TXT/?uri=CELEX%3A52016SC0017

Table 2: Assumptions on future dental amalgam demand in the baseline scenario

Group	Share of dental amalgam in 2010 (in % restorations)	Expected share of dental amalgam in 2025 (in % restorations)	Dental Hg use in 2010 (t)	Projected dental Hg use in 2025 (t)	Comments
**Group 1**<br>**DK, EE, SE, IT, FI**	0-5%	0%	0.3-0.4	0	This group includes countries where amalgam use is very limited and is expected to cease in the mid-term due to policy measures in place (e.g. SE) or other factors.
**Group 2**<br>**BG, BE, CY, DE, HU, IE, LU, NL, PT, ES, LV**	6-35%	5 to 15%	9 – 12	3– 8	In these countries, demand for dental amalgam is expected to continue to decrease until it reaches a relatively low share of restorations.
**Group 3**<br>**AT, CZ, FR, GR, LT, MT, PL, RO, SK, SI, UK**	>35%	20-30%	46 - 78	23-35	This group includes countries where dental amalgam is still widely used, as well as less wealthy countries where a large proportion of the population may not be able to bear the additional cost of Hg-free restorations. In addition, due to the currently high use of dental amalgam in these countries, there would also be a high proportion of dentists unwilling to change their current practices.
		**EU-27**	55 - 95	27-43	

DATA from: data.euro.who.int

Perinatal deaths per 1000 births																												
Years	Austria	Belgium	Bulgaria	Cyprus	Czech Republic	Denmark	Estonia	Finland	France	Germany	Greece	Hungary	Ireland	Italy	Latvia	Lithuania	Luxembourg	Malta	Netherlands	Poland	Portugal	Romania	Slovakia	Slovenia	Spain	Sweden	United Kingdom
1999	3.79	4.87	12.95	…	4.72	6.22	7.83	3.16	6.55	3.91	8.77	5.39	5.95	6.66	9.97	6.69	5.36	9.92	9.46	7.55	5.46	11.83	7.17	4.89	5.68	4.57	8.23
2000	3.87	5.01	12.16	…	4.01	5.8	6.37	3.67	6.58	6.07	7.88	5.74	6.29	5.63	9.06	8.27	6.78	4.6	9.05	6.68	5.07	12.1	6.57	4.09	5.51	4.68	8.15
2001	3.55	4.55	12.32	…	3.78	5.48	5.79	3.29	6.91	5.94	8	5.7	6.06	5.67	8.82	5.72	4.49	5.09	9.19	6.34	4.72	11.77	6.48	4.54	5.6	4.44	8.02
2002	3.43	4.57	12.57	…	3.97	4.84	6	3.37	9.99	5.83	7.22	5.38	6.29	5.44	10.65	7.29	4.8	6.65	8.83	5.75	4.9	11.76	6.39	5.15	5.24	3.96	8.3
2003	3.61	4.61	12.1	…	3.65	4.85	5.84	3.4	10.77	5.91	6.58	5.34	6.31	4.66	7.75	6.01	4.5	4.72	8.34	5.6	4.25	11.79	6.72	4.34	4.93	3.87	8.46
2004	3.2	4.42	12.21	…	3.58	4.7	5.16	2.91	10.87	5.89	6.28	4.8	5.54	4.55	7.8	6.27	4.16	5.91	5.04	5.63	3.63	12.24	5.82	4.16	4.87	3.9	8.16
2005	3.38	4.12	12.02	…	3.49	4.82	6.07	3.02	10.47	5.55	5.66	5.01	5.25	4.56	8.11	5.42	3.46	3.12	5.21	5.27	3.64	10.57	5.47	5.2	4.91	3.34	7.95
2006	3.2	4.18	10.69	…	3.63	3.77	4.17	3.06	10.92	5.55	4.94	4.86	5.05	4.45	7.39	5.74	2.98	1.8	4.62	4.96	3.98	9.83	5.58	3.49	4.78	3.59	7.89
2007	3.1	4.21	10.97	4.19	3.58	4.12	4.13	3.33	10.75	5.52	5.32	4.92	5.19	4.28	6.53	5.58	3.21	4.12	4.3	4.89	3.79	8.84	5.33	3.89	4.47	3.46	7.72
2008	2.96	4.01	10.54	…	2.93	3.47	4.26	2.91	12.05	5.3	4.4	4.32	4.8	4.45	6.29	5.3	3.65	6.91	4.19	4.72	3.27	7.96	5.35	3.81	4.37	3.42	7.54
2009	3.02	3.96	11.3	…	3.24	2.83	4.51	2.84	13.35	5.33	5.5	4.68	5.11	4.61	7.04	4.96	4.54	6.02	4.27	4.02	3.82	7.33	5.11	3.7	4.53	3.35	7.6
2010	3.01	4.06	11.38	…	3.08	3.03	3.17	2.52	11.73	5.35	5.86	4.12	4.79	…	5.74	4.41	3.38	6.22	4.07	4.24	2.93	7.04	4.62	2.71	4.42	3.01	7.39
2011	3.09	4.04	11.69	4.87	3.63	2.88	3.14	2.52	11.04	5.47	5.37	4.16	4.21	4.35	7.09	4.74	3.97	7.69	4.13	4.15	3.16	6.88	4.36	3.23	4.59	3.2	7.48
2012	2.96	4.24	10.95	5.73	3.63	3.21	3.07	2.63	11.58	5.29	5.64	4.03	4.19	4.44	6.03	3.94	4.66	3.88	3.66	4.14	3.55	6.22	4.7	2.4	4.64	3.17	6.99
2013	3.13	3.78	10.29	6.04	2.93	3.26	2.37	2.19	11.71	5.45	5.8	3.85	4.56	…	4.88	4.32	3.98	4.72	3.76	3.74	4.16	6.49	4.16	3.27	4.59	5.16	6.69
2014	2.88	…	10.41	2.96	3.1	3.34	3.25	2.71	11.76	5.45	…	4.23	6.39	…	4.84	4.38	2.88	5.72	…	3.45	5.14	6.62	4.58	2.22	4.57	5.14	…
2015	…	…	…	…	…	2.69	…	…	…	…	…	…	…	…	…	…	…	…	…	…	…	…	…	…	…	…	…
