# Supplementary material for: Perinatal death and exposure to dental amalgam fillings during pregnancy in the population-based MoBa cohort
Source: PLoS One. 2018 Dec 7;13(12):e0208803. doi: 10.1371/journal.pone.0208803 (PMC6286137; doi:10.1371/journal.pone.0208803)
Supplement: S2 File — (PDF) [file pone.0208803.s002.pdf]

S2 File. References to Questionnaire 1 and Questionnaire 3.

Questionnaire 1 (sent out in the 15th week of pregnancy)

English version (downloaded 30.10.2018): https://www.fhi.no/globalassets/dokumenterfiler/studier/moba/dokumenter/questionnaire---week-15-of-pregnancy-to-mother.pdf

Questions used: Number 27, 28, 50, 95, and 108.

Norwegian version (downloaded 30.10.2018): https://www.fhi.no/globalassets/dokumenterfiler/studier/moba/dokumenter/mor-og-barn-sporreskjema-1-15.-svangerskapsuke-versjon-1e.pdf

Questions used: Number 27, 28, 50, 95, and 108.

Questionnaire 3 (sent out in 30th week of pregnancy)

English version (downloaded 30.10.2018): https://www.fhi.no/globalassets/dokumenterfiler/studier/moba/dokumenter/questionnaire-3---30-weeks-of-gestation.pdf

Question used: Number 36

Norwegian version (downloaded 30.10.2018): https://www.fhi.no/globalassets/dokumenterfiler/studier/moba/dokumenter/mor-og-barn-sporreskjema-3-30.svangerskapsuke-versjon-3e.pdf

Question used: Number 39
