# Supplementary material for: Perinatal death and exposure to dental amalgam fillings during pregnancy in the population-based MoBa cohort
Source: PLoS One. 2018 Dec 7;13(12):e0208803. doi: 10.1371/journal.pone.0208803 (PMC6286137; doi:10.1371/journal.pone.0208803)
Supplement: S1 Table — (PDF) [file pone.0208803.s003.pdf]

S1 Table. EU countries divided into groups of estimated proportion of amalgam fillings placed in 2010.

Group 1 countries (estimated proportion 0-5%)	Group 2 countries (estimated proportion 6-35%)	Group 3 countries (estimated proportion >35%)
Denmark	Belgium	Austria
Estonia	Bulgaria	Czech Republic
Finland	Cyprus [b]	France
Italy [a]	Germany	Greece
Sweden	Hungary	Lithuania
	Ireland	Malta
	Latvia	Poland
	Luxembourg	Romania
	Netherlands	Slovakia
	Portugal	Slovenia
	Spain	United Kingdom

Data from Bio Intelligence Service [11].
[a] Missing data for perinatal mortality in 2010
[b] Excluded in Fig 3 because of missing data
